# Supplementary figures and images for: Kynurenine-3-monooxygenase (KMO) broadly inhibits viral infections via triggering NMDAR/Ca2+ influx and CaMKII/ IRF3-mediated IFN-β production
Source: PLoS Pathog. 2022 Mar 2;18(3):e1010366. doi: 10.1371/journal.ppat.1010366 (PMC8920235; doi:10.1371/journal.ppat.1010366)

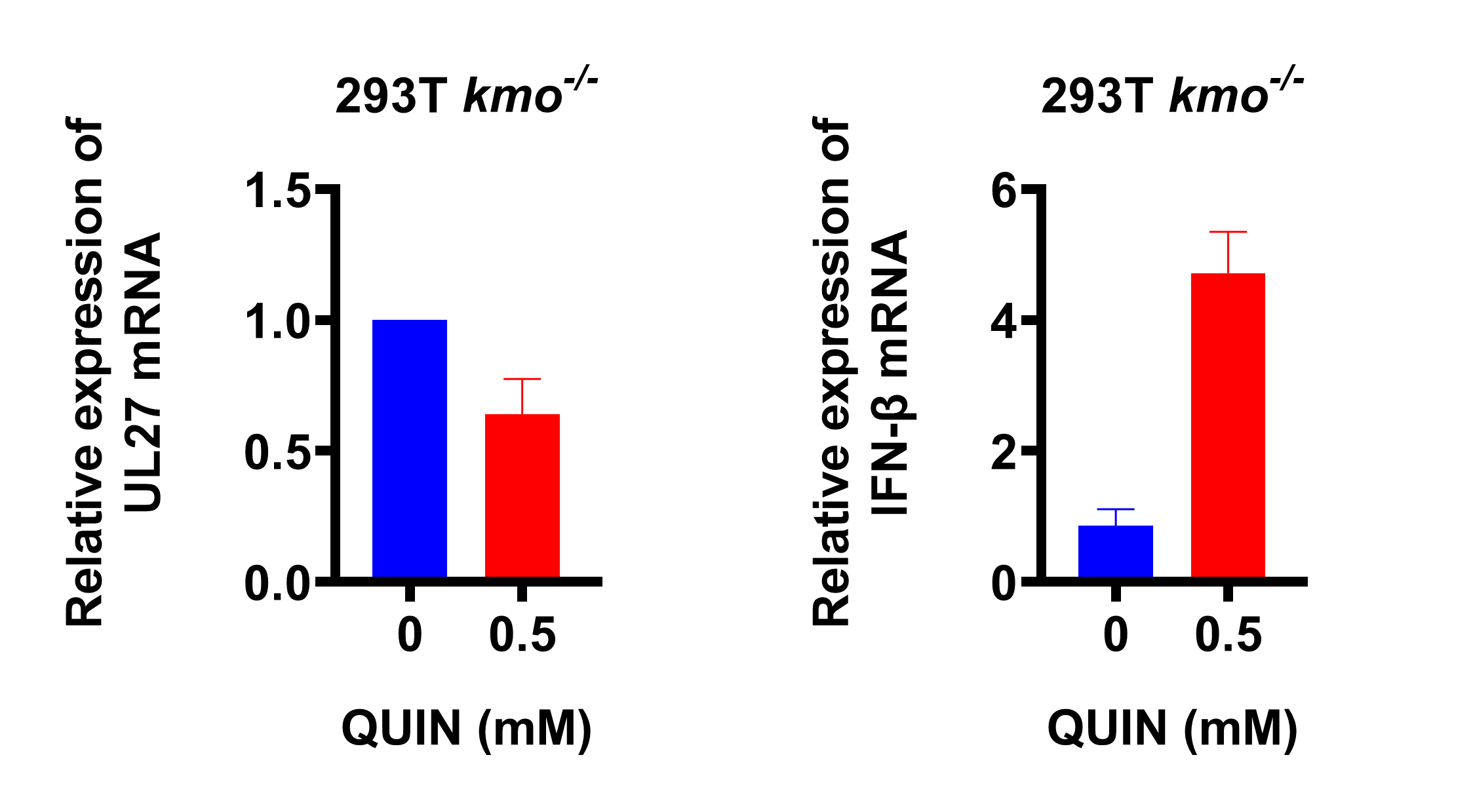

Supplement: S1 Fig — 293T kmo-/- cell lines were pretreated with QUIN for 8 h and then infected with HSV-1 at MOI of 0.25 for 8 h. Then, the expressions of HSV-1 and IFN-β were measured by RT-qPCR. The expression level of mRNA was normalized to the expression of β-actin, and the data from at least triplicates were shown as the mean ± SD. *P < 0.05, **P < 0.01, ***P < 0.001. (TIF) [file ppat.1010366.s001.tif]

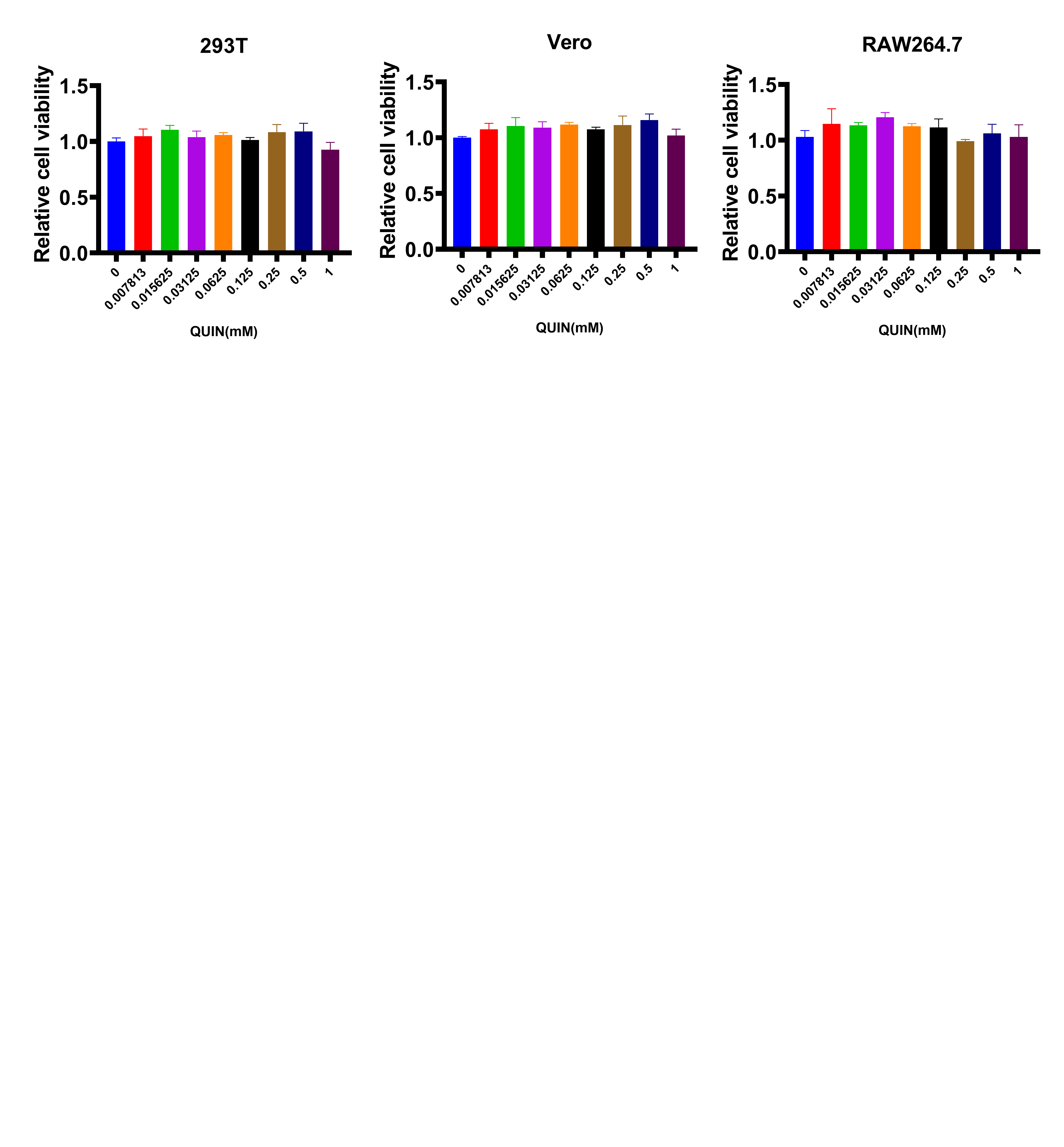

Supplement: S2 Fig — The different concentrations of QUIN were added into 293T, Vero, and Raw264.7 cells for 72 hours, and then the cell viability was detected with CCK8 assay. The final data are presented as the mean ± SD of at least triplicate experiments. (TIF) [file ppat.1010366.s002.tif]

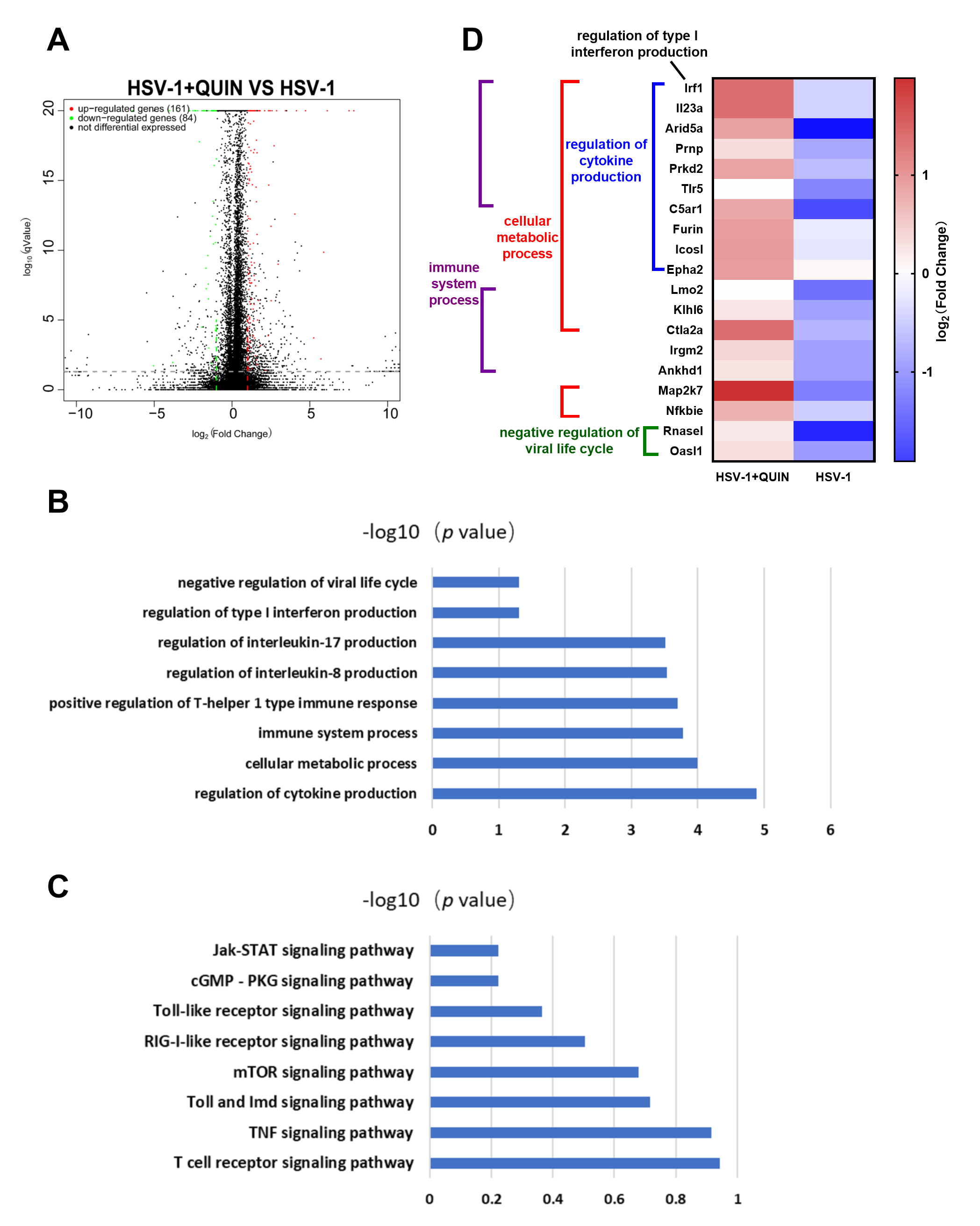

Supplement: S3 Fig — (A) Representative of the volcano plots to identify the differential gene expression (DGE) between QUIN-treated cells and non-QUIN-treated cells. Adjust P-value < 0.05, fold change |FC| > 1.5. Red dots represent those up-regulated genes. Green dots represent those down-regulated genes. Black dots represent those non-changed genes (Non-DEG). (B) The gene ontology (GO) annotation analysis for the related DEGs involved in QUIN treatment. (C) The enrichment analysis of Kyoto encyclopedia of genes and genomes (KEGG) of the related signaling pathways by QUIN treatment (P-value < 0.05). (D) Heatmap of the 19 selected genes involved in antiviral signaling pathway by QUIN treatment (P-value < 0.05). (TIF) [file ppat.1010366.s003.tif]

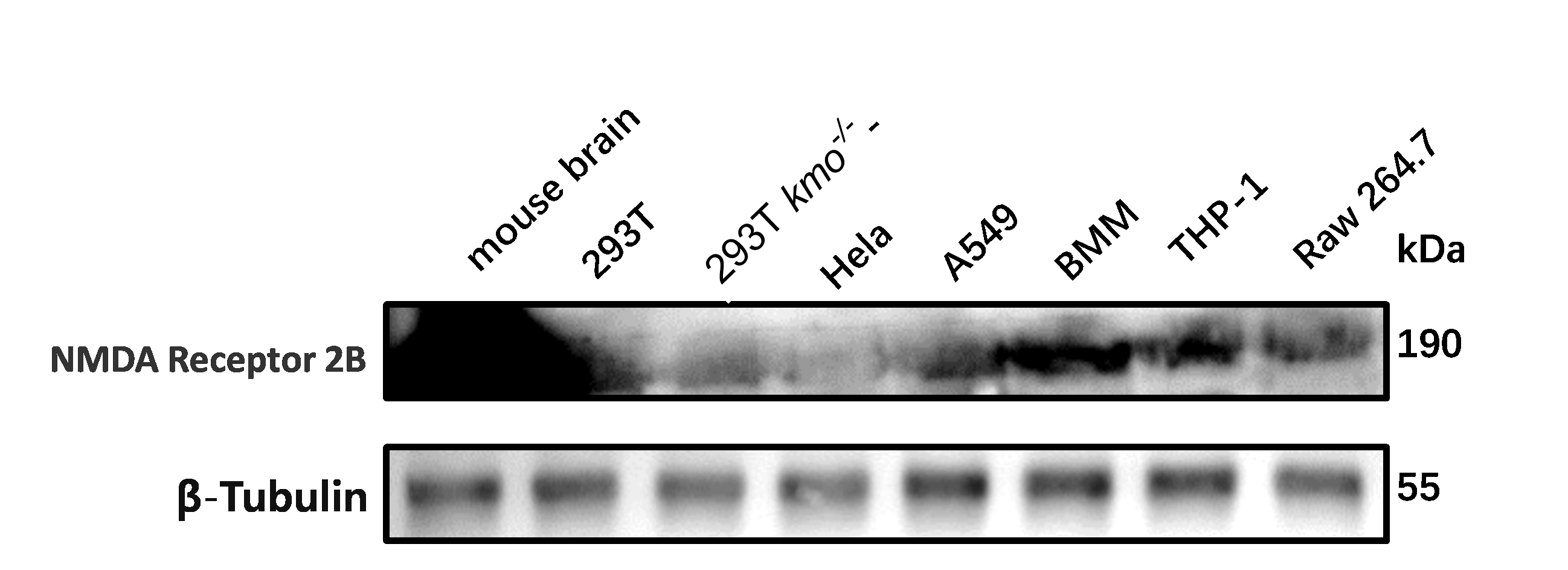

Supplement: S4 Fig — The Western Blotting analysis confirmed NMDAR protein expression in 293T, 293T kmo-/-, Hela, A549, BMM, THP-1, Raw 264.7 cell lines. Mouse brain protein lysate as the positive control. (TIF) [file ppat.1010366.s004.tif]

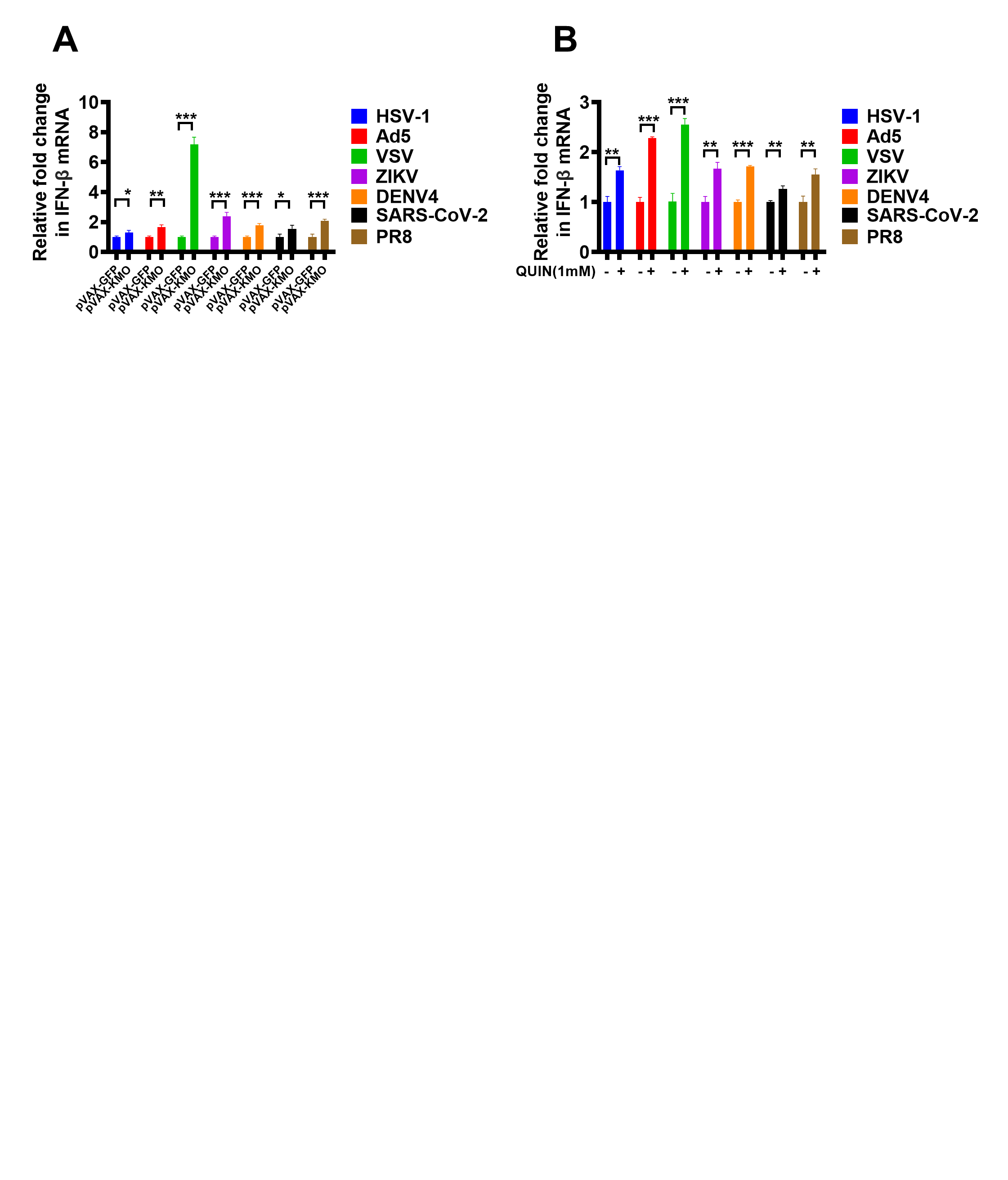

Supplement: S5 Fig — (A) Cells were transfected with different concentrations of KMO-expressing plasmid for 24 h, followed by viral infections for 8 h at MOI of 1, including HSV-1, VSV, ZIKV, DENV4, Ad5, SARS-CoV-2 pseudovirus, and PR8, and then the expression of IFN-β was quantified by RT-qPCR. (B) Cells were pretreated with QUIN at different concentrations for 8 h, followed by viral infections for 8 h at MOI of 1, including HSV-1, VSV, ZIKV, DENV4, Ad5, SARS-CoV-2 pseudovirus, and PR8, and then the expression of IFN-β was quantified by RT-qPCR. The expression level of mRNA was normalized to the expression of β-actin, and the data from at least triplicates were shown as the mean ± SD. *P < 0.05, **P < 0.01, ***P < 0.001. (TIF) [file ppat.1010366.s005.tif]

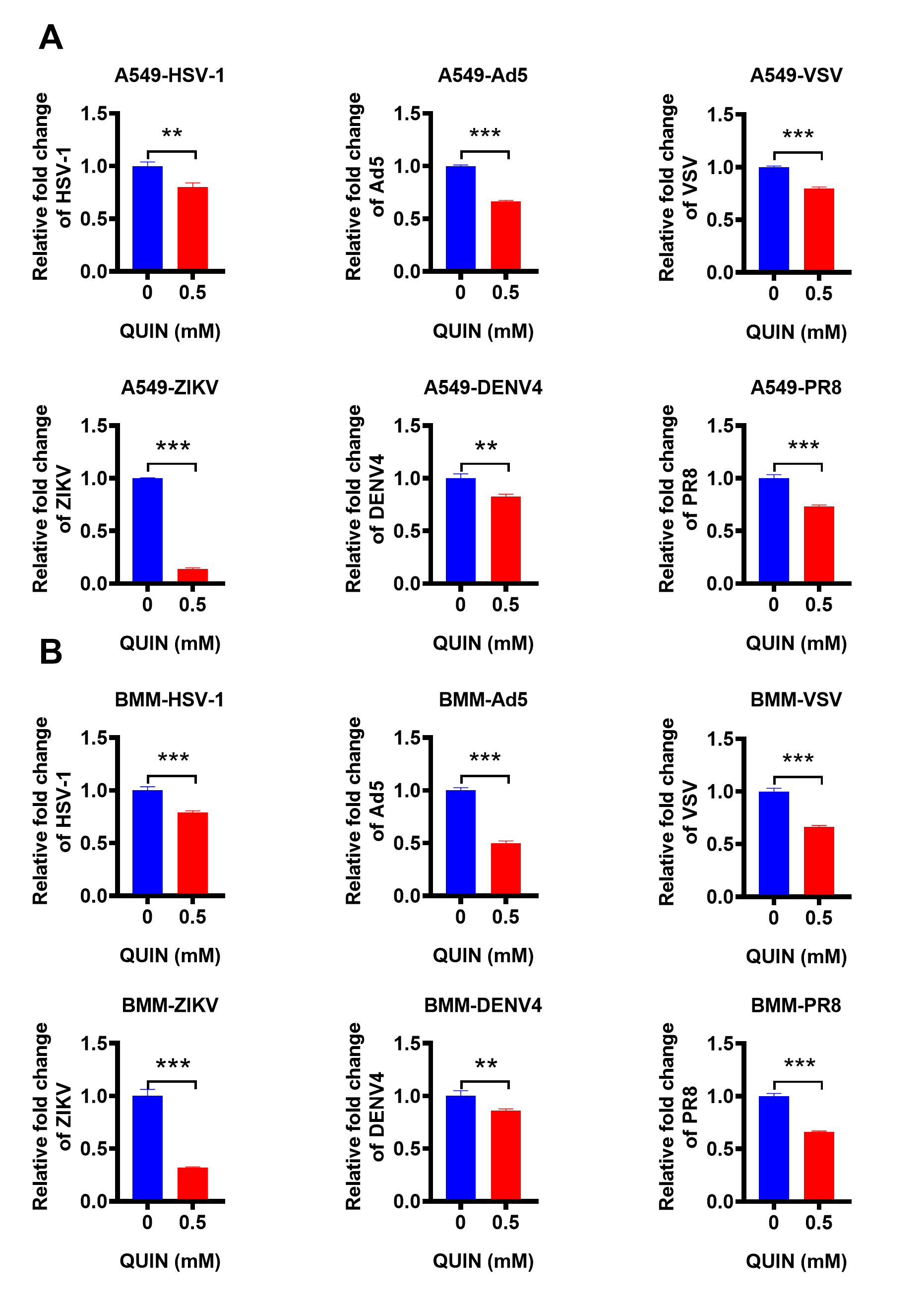

Supplement: S6 Fig — A549 cell lines and BMM cell lines were pretreated with QUIN (1mM) for 8 h, followed by infected with different viruses at MOI of 1 for 8 h. The expression of viruses was measured by RT-qPCR. The expression level of mRNA was normalized to the expression of β-actin, and the data from at least triplicates were shown as the mean ± SD. *P < 0.05, **P < 0.01, ***P < 0.001. (TIF) [file ppat.1010366.s006.tif]

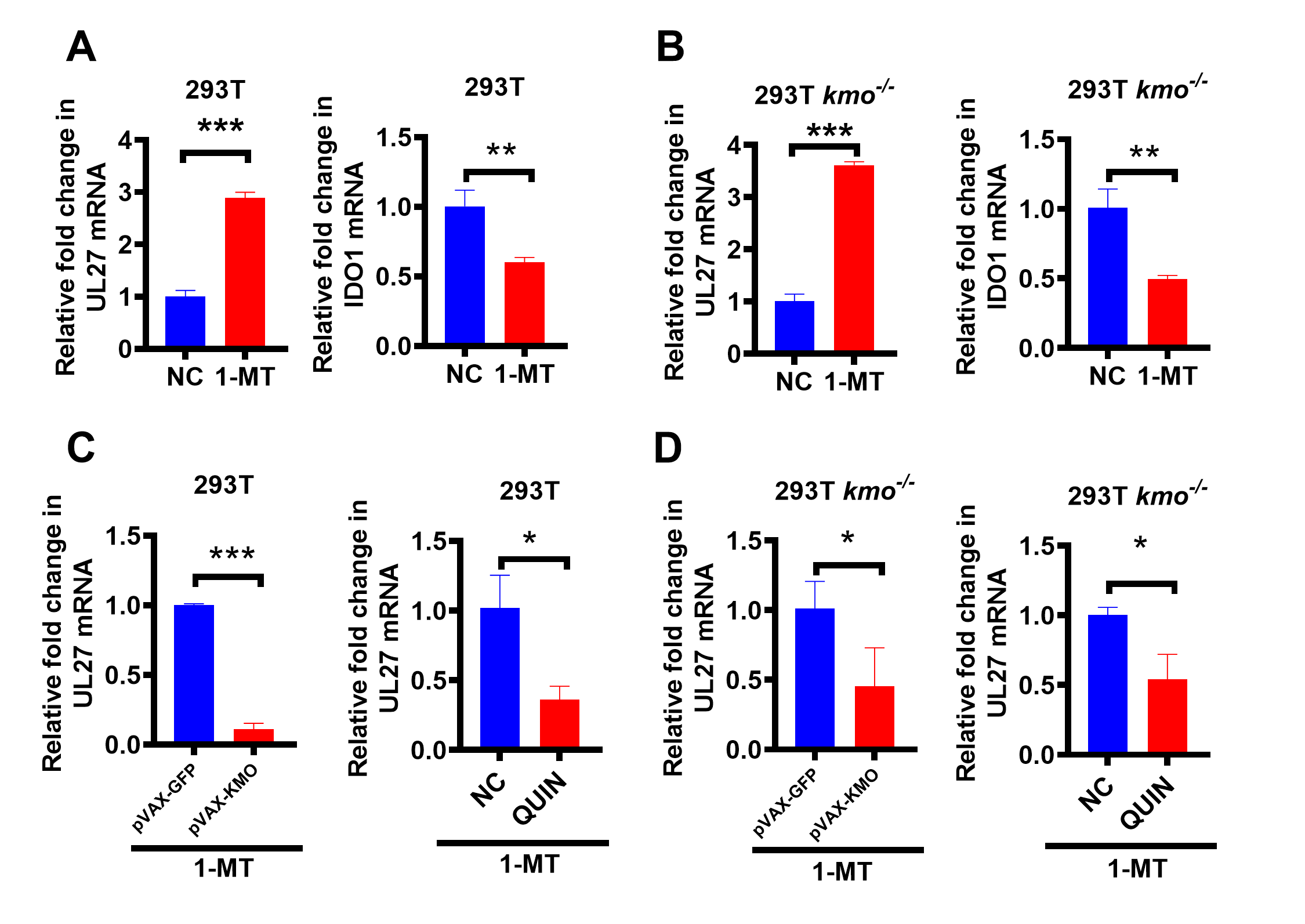

Supplement: S7 Fig — (A,B) 293T cell lines were pretreated with IDO1 inhibitor 1-MT (10 μM) for 4 h, followed by HSV-1 infection at MOI of 1 for 8 h. (C,D) Cells were pretreated with 1-MT, and then treated with KMO over-expression or QUIN followed by HSV-1 infection. The expression of HSV-1 and IDO1 were measured by RT-qPCR. The expression level of mRNA was normalized to the expression of β-actin, and the data from at least triplicates were shown as the mean ± SD. *P < 0.05, **P < 0.01, ***P < 0.001. (TIF) [file ppat.1010366.s007.tif]

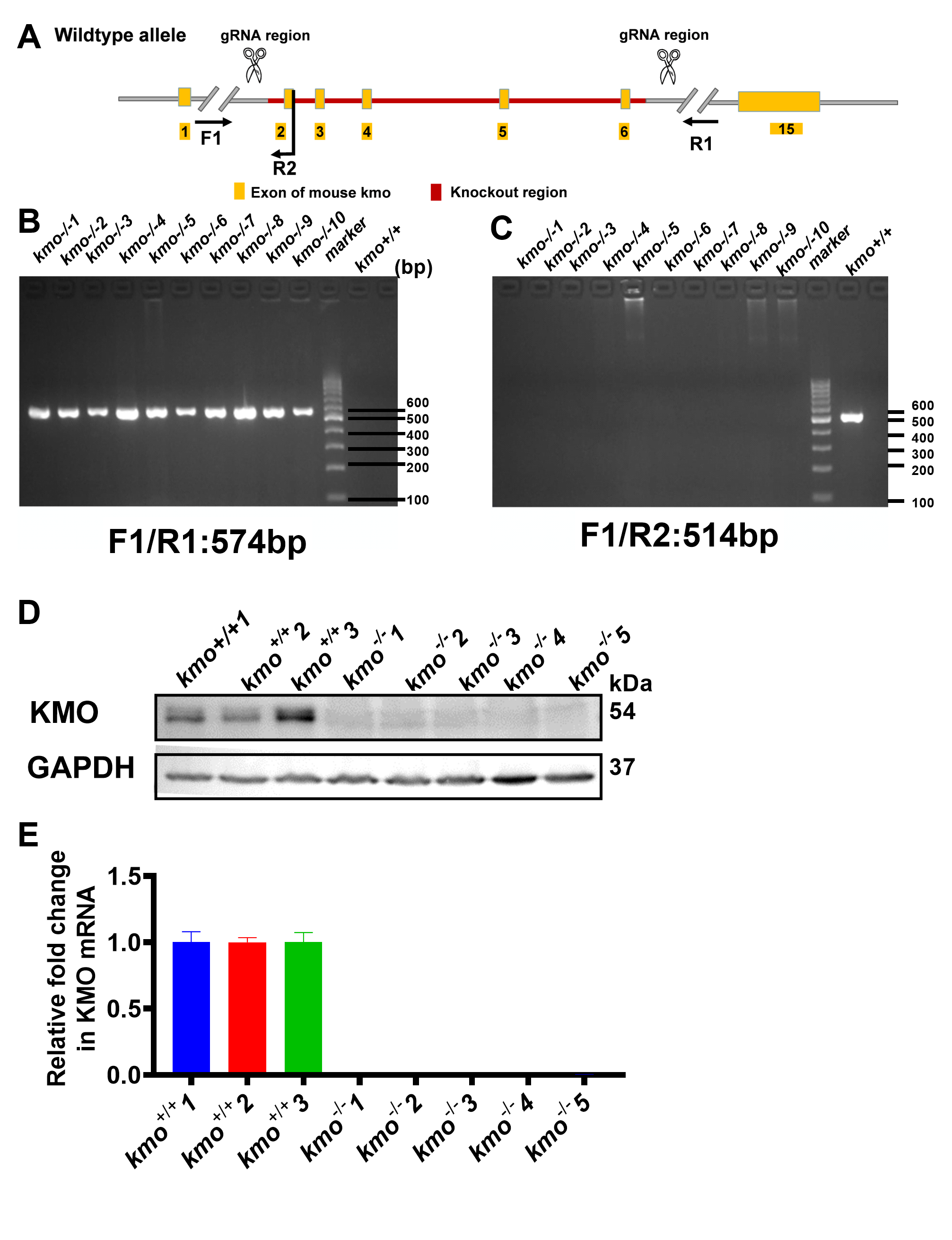

Supplement: S8 Fig — (A) kmo knockout strategy. The position shown by the scissors was the two sites of kmo exons targeted by designed CRISPR/Cas9. The red line represents the knocked-out fragment. F1/R1 primer pair and F1/R2 primer pair were the positions of the two primers to identify whether the knockout was successful. (B) The kmo-/- mice and wild-type mice were identified by F1/R1 primers. Theoretically, the amplified fragment of kmo-/- mice should be 574 bp (base pair) in length, while that of wild-type mice is too long to be amplified. (C) The kmo-/- mice and wild-type mice were identified by F1/R2 primers. Theoretically, the amplified fragment of wild-type mice should be 514 bp (base pair) in length, while that of kmo-/- mice cannot be amplified because of the lack of R2 sequence. (D) The kmo-/- mice and wild-type mice were identified by Western Blotting (top) and RT-qPCR (bottom). The expression level of mRNA was normalized to the expression of β-actin, and the data from at least triplicates were shown as the mean ± SD. *P < 0.05, **P < 0.01, ***P < 0.001. (TIF) [file ppat.1010366.s008.tif]

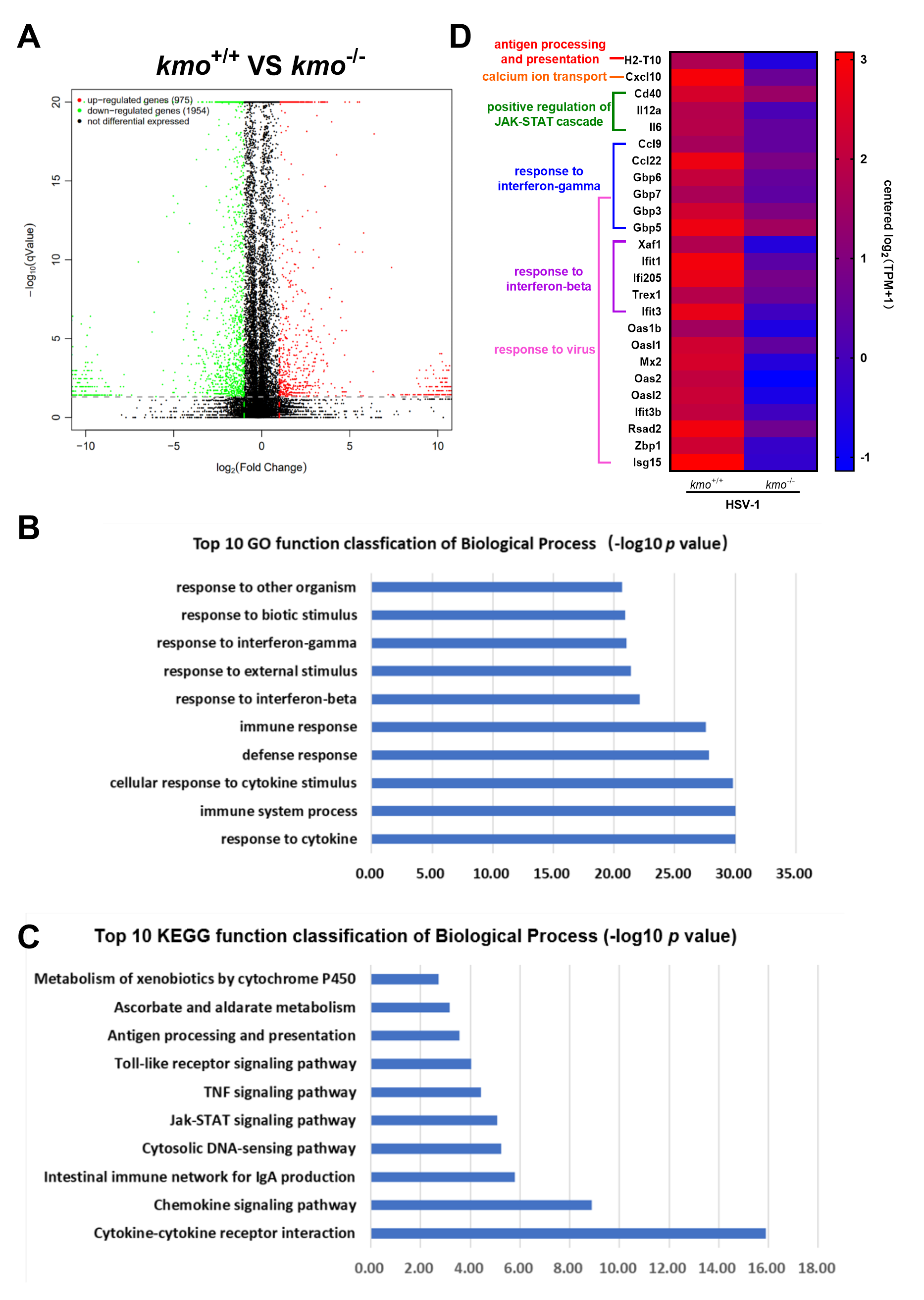

Supplement: S9 Fig — (A) Representative of the volcano plots to identify the differential gene expression (DGE) between bone marrow-derived macrophages from wild kmo+/+ mice and kmo-/- mice. Adjust P-value < 0.05, fold change |FC| > 1.5. Red dots represent those up-regulated genes. Green dots represent those down-regulated genes. Black dots represent those non-changed genes (Non-DEG). (B) The gene ontology (GO) annotation analysis for the upregulated DEGs in bone marrow-derived macrophages from wild kmo+/+ mice compared to kmo-/- mice. (C) The enrichment analysis of Kyoto encyclopedia of genes and genomes (KEGG) of the upregulated signaling pathways in bone marrow-derived macrophages from wild kmo+/+ mice compared to kmo-/- mice (P-value < 0.05). (D) Heatmap of the selected genes involved in antiviral signaling pathway (P-value < 0.05). (TIF) [file ppat.1010366.s009.tif]

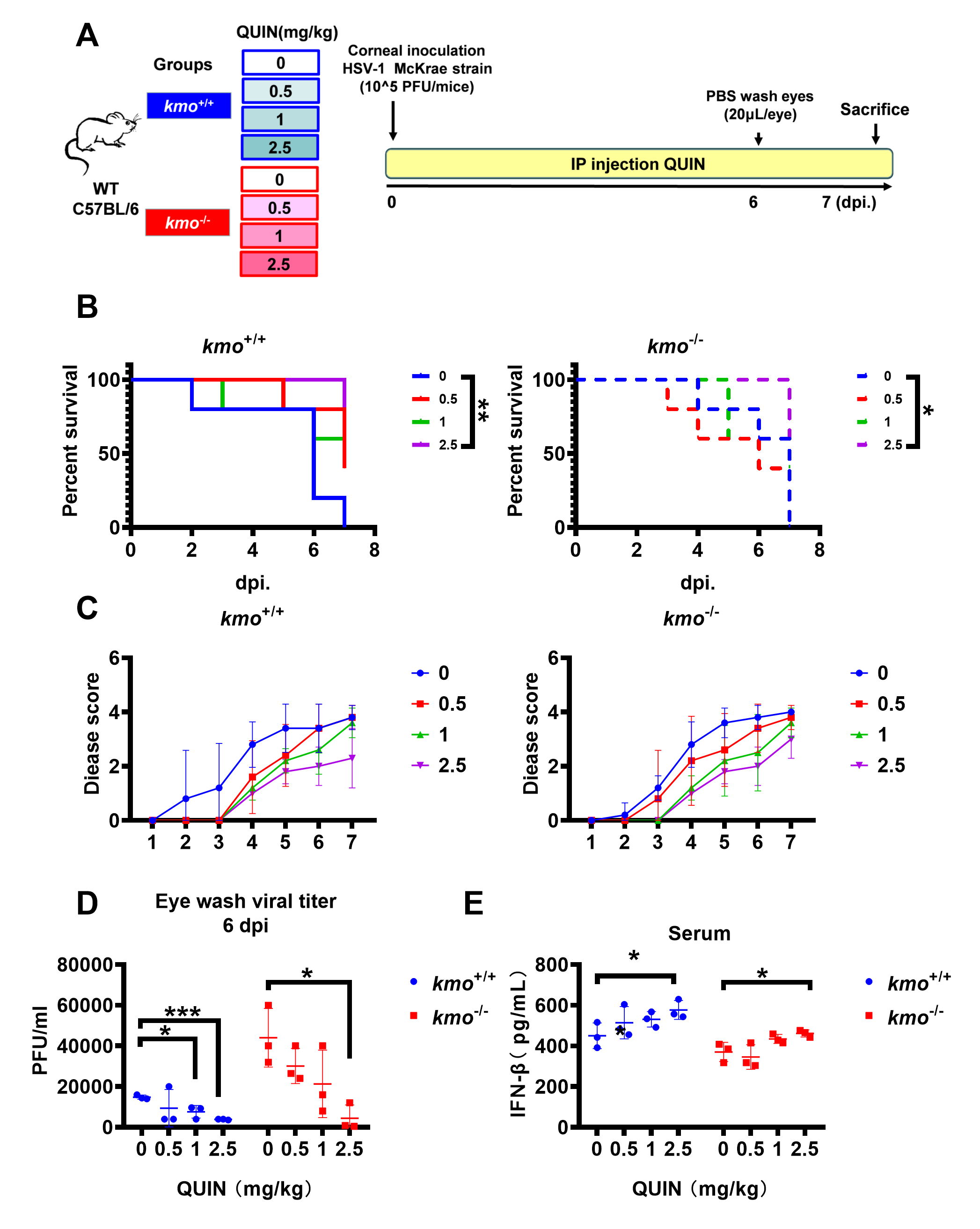

Supplement: S10 Fig — (A) Schedule for evaluating the rescue effect of QUIN on antiviral infections in kmo-/- mice. Briefly, both kmo+/+ C57BL/6 mice and kmo-/- C57BL/6 mice were corneally inoculated with 1×10^5^ PFU HSV-1 McKrae strain, disease symptoms of experimental mice were monitored until 7 dpi. (B,C) Survival curve and disease score of experimental mice in different groups over time post-infection (n = 5 per group). (D) The HSV-1 titer in the eye washing fluid at 6 dpi was measured by plaque assay (n = 3 per group). (E) The concentration of IFN-β in the serum of experimental mice at 7 dpi was measured by ELISA assay (n = 3 per group). The final data are presented as the mean ± SD of triplicate experiments. *P < 0.05, **P < 0.01, ***P < 0.001. (TIF) [file ppat.1010366.s010.tif]

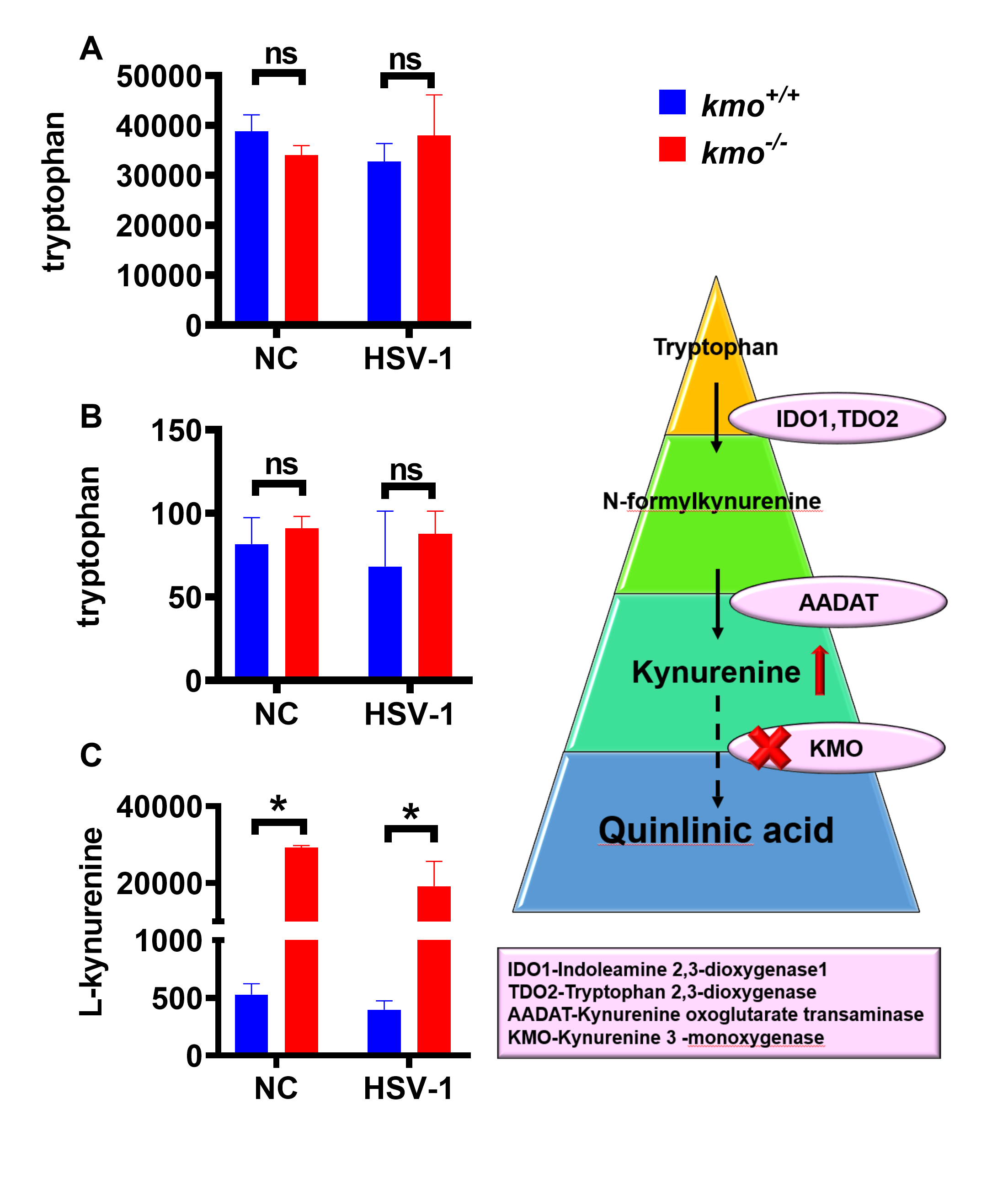

Supplement: S11 Fig — The blood of virus-infected and non-virus-infected kmo+/+ mice and kmo-/- mice were collected by orbital bleeding, and the plasma was separated to determine tryptophan metabolites including Trytophan (A), N-formyl-kynurenine (B), Kynurenine (C). The final data are presented as the mean ± SD. *P < 0.05, **P < 0.01, ***P < 0.001. (TIF) [file ppat.1010366.s011.tif]
