# Supplementary material for: Kynurenine-3-monooxygenase (KMO) broadly inhibits viral infections via triggering NMDAR/Ca2+ influx and CaMKII/ IRF3-mediated IFN-β production
Source: PLoS Pathog. 2022 Mar 2;18(3):e1010366. doi: 10.1371/journal.ppat.1010366 (PMC8920235; doi:10.1371/journal.ppat.1010366)
Supplement: S1 Table — (DOCX) [file ppat.1010366.s012.docx]

**S1 Table The sequences of siRNA, sgRNA, shRNA used in this study.**

| Gene | Sequence (5’-3’) |
| --- | --- |
| Homo-siKMO | GGAGCCTATTCAACTGTCA |
| Homo-sgCDS1-F | CACCGCGAGTGGCTACCTTCACACG |
| Homo-sgCDS1-R | AAACCGTGTGAAGGTAGCCACTCGC |
| Homo-sgCDS2-F | CACCGAGCTAGGGAAGATACTCGAG |
| Homo-sgCDS2-R | AAACCTCGAGTATCTTCCCTAGCTC |
| Homo-sgEXON1-F | CACCGCCCTGACTAAACATTGCCGC |
| Homo-sgEXON1-R | AAACGCGGCAATGTTTAGTCAGGGC |
| Homo-sgEXON2-F | CACCGCCAGCGGCAATGTTTAGTC |
| Homo-sgEXON2-R | AAACGACTAAACATTGCCGCTGGC |
| Homo-shKMO1-F | CCGGCCACAGGCTGTTGAAATGTAACTCGAGTTACATTTCAACAGCCTGTGGTTTTTG |
| Homo-shKMO1-R | AATTCAAAAACCACAGGCTGTTGAAATGTAACTCGAGTTACATTTCAACAGCCTGTGG |
| Homo-shKMO2-F | CCGGGAGAGATTTCTTCATGCGATTCTCGAGAATCGCATGAAGAAATCTCTCTTTTTG |
| Homo-shKMO2-R | AATTCAAAAAGAGAGATTTCTTCATGCGATTCTCGAGAATCGCATGAAGAAATCTCTC |
| Homo-shKMO3-F | CCGGCCACCTAAGAACGGAGATTATCTCGAGATAATCTCCGTTCTTAGGTGGTTTTTG |
| Homo-shKMO3-R | AATTCAAAAACCACCTAAGAACGGAGATTATCTCGAGATAATCTCCGTTCTTAGGTGG |
| Mus-siKMO | CCUUCCAAACAUGGACAAATT |
| Mus-sgCDS1-F | CACCGTCCAGCACATCGCTGCGCGT |
| Mus-sgCDS1-R | AAACACGCGCAGCGATGTGCTGGAC |
| Mus-sgCDS2-F | CACCGCGCGTGGCTAAATCTGCACG |
| Mus-sgCDS2-R | AAACCGTGCAGATTTAGCCACGCGC |
| Mus-shKMO1-F | CCGGCTTTGATTACACTCAGCAATACTCGAGTATTGCTGAGTGTAATCAAAGTTTTTG |
| Mus-shKMO1-R | AATTCAAAAACTTTGATTACACTCAGCAATACTCGAGTATTGCTGAGTGTAATCAAAG |
| Mus-shKMO2-F | CCGGGCCTATATGATGATCGCCCTTCTCGAGAAGGGCGATCATCATATAGGCTTTTTG |
| Mus-shKMO2-R | AATTCAAAAAGCCTATATGATGATCGCCCTTCTCGAGAAGGGCGATCATCATATAGGC |
| MUS-SICaMKII | CACCACCATTGAGGACGA |
