# Supplementary material for: Kynurenine-3-monooxygenase (KMO) broadly inhibits viral infections via triggering NMDAR/Ca2+ influx and CaMKII/ IRF3-mediated IFN-β production
Source: PLoS Pathog. 2022 Mar 2;18(3):e1010366. doi: 10.1371/journal.ppat.1010366 (PMC8920235; doi:10.1371/journal.ppat.1010366)
Supplement: S2 Table — (DOCX) [file ppat.1010366.s013.docx]

**S2 Table Primers for RT-qPCR used in this study.**

| Gene | Sequence (5’-3’) |
| --- | --- |
| HSV-1-*UL-27*-F | GCCTTCTTCGCCTTTCGC |
| HSV-1*UL-27*-R | CGCTGTGCCCTTCTTCTT |
| vsv-f | TTGGCAAGTATGCTAAGTCAG |
| vsv-r | AGGACTTGAGATACTCACGAA |
| Ad5-f | GAGTTGGCACCCCTATTCGA |
| Ad5-r | GTTGCTGTGGTCGTTCTGGT |
| DENV4-f | TCAATACCGAACCTGAAGACA |
| DENV4-r | GACCAGCATCATTAGGACAAA |
| ZIKV-f | TTGGTCATGATACTGCTGATTGC |
| ZIKV-f-r | CCTTCCACAAAGTCCCTATTGC |
| PR8 NP-f | AGGACAAGAGCTCTTGTTCG |
| PR8 NP-r | CTCTTGTGTGCTGGATTCTC |
| SARS-CoV-2 N-F | TCTACGCAGAAGGGAGCA |
| SARS-CoV-2 N-R | AGCAAGAGCAGCATCACC |
| Homo- β-actin-F | CATGTACGTTGCTATCCAGGC |
| Homo- β-actin-R | CTCCTTAATGTCACGCACGAT |
| homo&mus-kmo-f | TGTGATGGAGCCTATTCAA |
| homo&mus-kmo-r | GATATCATGGGCTGGGCAGG |
| Homo-IFN-β-F | TCCTACAAAGAAGCAGCAA |
| Homo-IFN-β-R | TCCTCAGGGATGTCAAAGT |
| Mus-β-actin-F | CGTTGACATCCGTAAAGACC |
| Mus-β-actin-R | TAGGAGCCAGAGCAGTAATC |
| Mus-IFN-β-F | TCACCTACAGGGCGGACTTC |
| Mus-IFN-β-R | TCTCTGCTCGGACCACCATC |
| IDO1-F | TGGCAAACTGGAAGAAAAAGG |
| IDO1-R | TGGCCTGCACTGCCCCCTG |
