# Supplementary material for: Kynurenine-3-monooxygenase (KMO) broadly inhibits viral infections via triggering NMDAR/Ca2+ influx and CaMKII/ IRF3-mediated IFN-β production
Source: PLoS Pathog. 2022 Mar 2;18(3):e1010366. doi: 10.1371/journal.ppat.1010366 (PMC8920235; doi:10.1371/journal.ppat.1010366)
Supplement: S3 Table — (DOCX) [file ppat.1010366.s014.docx]

**S3 Table The sequence of HSV-1 peptide used in this study.**

| Name | Sequence (N’-C’) |
| --- | --- |
| HSV-gB-1 | SSIEFARL |
| HSV-gB-2 | RMLGDVMAV |
| HSV-gB-4 | RFADIDTVIHA |
| HSV-gB-5 | EEYAYSHQL |
| HSV-gB-6 | RYMALVSAM |
| HSV-gB-7 | YYLANGGFL |
| HSV-gB-8 | APYKFKATM |
| HSV-gB-9 | VGHRRYFTF |
| HSV-gB-10 | REMIRYMALVSAME |
| HSV-gB-11 | EMIRYMALVSAMER |
| HSV-gD-1 | SLKMADPNRFRGKDLP |
| HSV-gD-2 | DPEDSALL |
| HSV-gD-3 | KYALADASLKMADPNRFRGKDLP |
| HSV-gD-4 | KYALVDASL |
| HSV-gD-5 | NYYDSFSAV |
| HSV-gD-6 | KAPYTSTLL |
| HSV-gD-7 | VAPQIPPNW |
| HSV-gD-8 | TPNATQPEL |
| HSV-gD-9 | CGIVYWMRRHTQKA |
| HSV-gD-10 | GIVYWMRRHTQKAPK |
| HSV-other-1 | QTFDFGRL |
| HSV-other-2 | GDEYDDAADAAGDRAP |
| HSV-other-3 | LGQPEEGAPCQVVLQ |
| HSV-other-4 | DYATLGVGV |
