# Supplementary material for: Kynurenine-3-monooxygenase (KMO) broadly inhibits viral infections via triggering NMDAR/Ca2+ influx and CaMKII/ IRF3-mediated IFN-β production
Source: PLoS Pathog. 2022 Mar 2;18(3):e1010366. doi: 10.1371/journal.ppat.1010366 (PMC8920235; doi:10.1371/journal.ppat.1010366)
Supplement: S4 Table — (DOCX) [file ppat.1010366.s015.docx]

**S4 Table Key Resources** **used in this study.**

| REAGENT or RESOURCE | SOURCE | IDENTIFIER |
| --- | --- | --- |
| Antibodies | | |
| Anti-ICP27 antibody | Abcam | Cat# ab53480, RRID:AB_881581 |
| Anti-kynurenine 3-monooxygenase antibody | Abcam | Cat# ab130959, RRID:AB_11156090 |
| Recombinant Anti-GAPDH antibody (EPR16891) - Loading Control | Abcam | Cat# ab181602, RRID:AB_2630358 |
| Rabbit Anti-Mouse IgG - H&L Polyclonal Antibody, HRP Conjugated | Abcam | Cat# ab6728, RRID:AB_955440 |
| Goat Anti-Rabbit IgG - H&L Polyclonal antibody, Hrp Conjugated | Abcam | Cat# ab6721, RRID:AB_955447 |
| IRF-3 (D6I4C) XP antibod | Cell Signaling Technology | Cat# 11904, RRID:AB_2722521 |
| Phospho-IRF-3 (Ser396) (D6O1M) antibody | Cell Signaling Technology | Cat# 29047, RRID:AB_2773013 |
| Phospho-CaMKII (Thr286) (D21E4) Rabbit mAb antibody | Cell Signaling Technology | Cat# 12716, RRID:AB_2713889 |
| CaMKII-α (6G9) Mouse antibody | Cell Signaling Technology | Cat# 50049, RRID:AB_2721906 |
| NMDA Receptor 2B (GluN2B) (D8E10) Rabbit antibody | Cell Signaling Technology | Cat # 14544S  RRID:AB_2798506 |
| β-Tubulin Antibody | Cell Signaling Technology | Cat # 2146S  RRID:AB_823664 |
| Secondary Antibodies-Goat Anti-Rabbit lgG HRP | Abmart | Cat# M21007S  RRID: N/A |
| Secondary Antibodies-Goat Anti-Mouse lgG HRP | Abmart | Cat# M21005S  RRID: N/A |
| Viruses | | |
| HSV-GFP-Luc strain F virus (HSV-1) | Stored in our lab | N/A |
| VSV-GFP（VSV） | Gift from Dr. Tian Lan | VectorBuilder Biotechnology (Guangzhou) Co., Ltd. |
| DENV4 | Gift from Dr. Zhongyu Liu | Viral RNA switch mediates the dynamic control of flavivirus replicase recruitment by genome cyclization |
| ZIKV | Gift from Dr. Zhongyu Liu | A single substitution in the prM protein of Zika virus contributes to fetal microcephaly |
| Influenza virus A/PR8 | Gift from Prof. Yuelong Shu | Development of a new candidate H5N1 avian influenza virus for pre-pandemic vaccine production |
| Replication-competent SARS-CoV-2 virus-like-particles (SARS-CoV-2 GFP/ΔN) | Gift from Prof. [Qiang Ding](https://www.ncbi.nlm.nih.gov/pubmed/?term=Ding%20Q%5BAuthor%5D&cauthor=true&cauthor_uid=33658332) | A novel cell culture system modeling the  SARS-CoV-2 life cycle |
| HSV-1 strain McKrae (McKrae) | Gift from Prof. Jumin Zhou | HSV-1 infection and pathogenesis in the tree shrew eye following corneal inoculation |
| Ad5-GFP（Ad5） | Stored in our lab | N/A |
| Chemicals, peptides, and recombinant proteins | | |
| Lipopolysaccharide (LPS) | Invitrogen | 00-4976-93 |
| Poly(I:C) | Sigma | P1530-25MG |
| R848 | Stem cell | 73784 |
| [Recombinant Mouse IFN-alpha A Protein](https://www.rndsystems.com/cn/products/recombinant-mouse-ifn-alpha-a-protein_12100-1) | R&D Systems | NP_996753 |
| Quinaldic acid | Selleck | S6366 |
| KMO inhibitor (Ro 61-8048) | MCE | HY-12347 |
| NMDAR inhibitor ((-)-MK-801) | MCE | HY-15084B |
| IDO1 inhibitor 1-Methyl-D-tryptophan (1-MT) | Selleck | S7756 |
| Phorbol 12-myristate 13-acetate (PMA) | Selleck | S7791 |
| ionomycin (Ion) | Sigma | [200-664-3](https://www.sigmaaldrich.cn/CN/zh/search/200-664-3?focus=products&page=1&perPage=30&sort=relevance&term=200-664-3&type=egec_number) |
| Mouse 1× Lymphocyte Separation Medium | Dakewe Biotech Company Ltd., Shenzhen, China | 7211011/721101X |
| peptides | Genscript, Nanjing, China | N/A |
| Lipofectamine 2000 transfection reagent | Invitrogen | 11668019 |
| Lipofectamine RNAiMax transfection reagent | Invitrogen | 13778150 |
| Trizol Reagent | Thermo Fisher Scientific | 15596026 |
| [Calbryte™ 520 AM](https://www.aatbio.com/products/calbryte-520-am?upgrade=cal-520-am) | AAT Bioquest | 20651 |
| Hifair III 1st Strand cDNA Synthesis SuperMix for qPCR | Yeasen Biotechnology | 11141ES60 |
| PerfectStart SYBR Green qPCR supermix | TransGen Biotech | AQ601-02 |
| Critical commercial assays | | |
| Steady-Glo Luciferase Assay system | Promega | E2520 |
| *Fast* Mutagenesis System | TransGen Biotech | FM111-01 |
| Cell Counting Kit-8 (CCK8) | Yeasen | 40203ES60 |
| BCA Protein Assay Kit | Thermo Scientific | 23252 |
| Chemister High-sig ECL Western Blotting Substrate | Tanon | 180-5001 |
| Mouse IFN Beta ELISA Kit | Solarbio Life Science, | SEKM-0032 |
| Experimental models: Cell lines | | |
| 293T | ATCC | Cat#CRL-3216 |
| Vero | ATCC | Cat#CCL-81 |
| RAW 264.7 | Stored in our lab | N/A |
| Hela | Stored in our lab | N/A |
| A549 | Stored in our lab | N/A |
| THP-1 | Stored in our lab | N/A |
| *kmo-/-* 293T | This paper | N/A |
| WT-J2-BMM | Gift from Dr. [Genhong Cheng](https://pubmed.ncbi.nlm.nih.gov/?term=Cheng+G&cauthor_id=28314593) | Interferon-Inducible Cholesterol-25-Hydroxylase Broadly Inhibits Viral Entry by Production of 25-Hydroxycholesterol |
| Ifnar−/−J2-BMM | Gift from Dr. [Genhong Cheng](https://pubmed.ncbi.nlm.nih.gov/?term=Cheng+G&cauthor_id=28314593) | Interferon-Inducible Cholesterol-25-Hydroxylase Broadly Inhibits Viral Entry by Production of 25-Hydroxycholesterol |
| Caco-2-N | Gift from Prof. [Qiang Ding](https://www.ncbi.nlm.nih.gov/pubmed/?term=Ding%20Q%5BAuthor%5D&cauthor=true&cauthor_uid=33658332) | A novel cell culture system modeling the  SARS-CoV-2 life cycle |
| Experimental models: Organisms/strains | | |
| WT C57BL/6 mice | Laboratory Animal Resource Center of Sun Yat-sen University | N/A |
| kmo-/- C57BL/6 mice | Cyagen Biosciences | N/A |
| Recombinant DNA | | |
| pVAX-KMO | In house produced | N/A |
| pVAX-CH25H | In house produced | N/A |
| pVAX-GFP | In house produced | N/A |
| pLentiCRISPRv2 | Gift from Prof. Junjian Wang | Addgene #52961 |
| pMD2.G | Gift from Prof. Junjian Wang | Addgene #12259 |
| psPAX2 | Gift from Prof. Junjian Wang | Addgene #12260 |
| pLKO.1 vector | Gift from Prof. Junjian Wang | Addgene #10878 |
| Software and algorithms | | |
| GraphPad Prism8 | GraphPad Software | https://www.graphpad.com |
