# Supplementary material for: Kynurenine-3-monooxygenase (KMO) broadly inhibits viral infections via triggering NMDAR/Ca2+ influx and CaMKII/ IRF3-mediated IFN-β production
Source: PLoS Pathog. 2022 Mar 2;18(3):e1010366. doi: 10.1371/journal.ppat.1010366 (PMC8920235; doi:10.1371/journal.ppat.1010366)
Supplement: Attachment — Submitted filename: Response to reviewers.docx [file ppat.1010366.s016.docx]

**Point-by-point Responses to Reviewers' comments:**

**Part I - Summary**
*Reviewer #1: Exploring the novel antiviral agents is a public health priority against frequent outbreaks of emerging infectious diseases. In this manuscript, Zhao and colleagues reported that KMO and its enzymatic product QUIN exerted a novel broad-spectrum antiviral function in vitro and in vivo via triggering NMDAR/Ca2+ influx and CaMKII/ IRF3-mediated IFN-β production. Previously, KMO was recognized as a key rate-limiting enzyme in the Tryptophan metabolism, but its role in the innate immunity in response to viral infections has not been clarified. Thus, this work addresses an important topic to help determine the possibility of applying KMO as a novel target of antiviral drugs. The authors' intriguing data is therefore of considerable interest. There are still some issues to be addressed by the authors that would improve the manuscript.*

**Answer: Thank you for your kind comments and suggestions, which are very helpful in improving our manuscript. We performed additional experiments based on your suggestions, and we hope this version has been substantially improved.**

*Reviewer #2: This study showed that the mid-stream enzyme from the kynurenine pathway of the tryptophan metabolism, KMO, plays a role in modulating anti-viral activity. They showed that KMO and quinolinic acid (QA) can exert antiviral activity involving NMDA-induced calcium flux and IRF-mediated IFN-beta activity. They further showed that KMO expression and QA were responsible for inhibiting a broad spectrum of viruses that may confer a protective and susceptibility role in the host against viral infection.
A major strength of the study is that the authors used various approaches to examine the role of KMO and QA as novel antiviral targets. For this, I appreciate and acknowledge the efforts of this study. Another interesting result from this study that was understated in this study is the result of KMO and QA having a direct impact on the immune activity in driving antiviral activity.
A major concern I have is to what extent does IDO-1 involved in this study co-commitment with KMO activity? This is something that needs to be teased out since IDO-1 also has antiviral activity. For example, O Adams et al (J Virol, 2004 - https://dx.doi.org/10.1128/JVI.78.5.2632-2636.2004) showed that IDO-1 can exert antiviral effect via interferon activity. We know that viral infection can trigger IDO-1, which is likely to be the case in this study. Did the authors look at IDO-1 expression to make sure their result is due to KMO and not IDO-1? It is also possible that inhibition of KMO leads to accumulation of kynurenine which can have a negative feedback inhibition on IDO-1, thereby suppressing antiviral activity as well. Hence this is important missing info to look into.
In short, if the authors can prove that the antiviral effect is due to KMO and not IDO. This will be a novel role in the field of tryptophan research.*

**Answer: Thank you for your careful review and kind comments. Following your suggestions, we conducted additional experiments to clarify that KMO and QUIN have antiviral activities independent of IDO1 (S7 Fig, Line 211-217). In brief, we treated cells with 1-Methyl-D-tryptophan(1-MT), an inhibitor of IDO1. We found that 1-MT treatment weakened the antiviral effect by inhibiting IDO1.** **However, KMO and QUIN treatment could rescue the antiviral effect even after the IDO1 inhibition by 1-MT, indicating that the antiviral effects of KMO and QUIN were independent on IDO1. Thank you for your nice suggestions.**

*Reviewer #3: This study examined the mechanism of action of kynurenine-3-monooxygenase (KMO)-mediated antiviral effect. It was shown that the enzymatic product of KMO, quinolinic acid (QUIN), activates N-methyl-d-aspartate receptor (NMDAR) and Ca2+ influx, which subsequently activates Calcium/calmodulin29 dependent protein kinase II (CaMKII) / Interferon regulatory factor 3 (IRF3). Over-expression of KMO led to the inhibition of viral replication, while knockout or mutate KMO promote viral replication. Similarly, QUIN treatment led to the inhibition of viral replication of multiple viruses in cell culture. QUIN was also shown to have in vivo antiviral efficacy in HSV-1 infection mouse model.*

**Answer: Thank you for your kind comments.**

**Part II – Major Issues: Key Experiments Required for Acceptance**
*Reviewer #1: 1. The author analyzed the difference in transcriptomics of cell lines after virus infections with or without QUIN incubation and found the changes in the interferon pathway. It is recommended to analyze further the difference in transcriptomics of primary cells between KMO-/- mice and wild KMO+/+ mice after viral infections.*

**Answer: Thank you for your careful reading and comments. Following your suggestions, we further analyzed the difference in transcriptomics of bone marrow-derived macrophages from *kmo*^-/-^ mice and *kmo*^+/+^ mice.**

**In brief, compared to the *kmo*^-/-^ group, the gene ontology (GO) analyses showed that the *kmo*^+/+^ group upregulated the expression of multiple genes related to IFN-β and IFN-γ production immune system process, defense response. KEGG analyses revealed enrichment in the Jak-STAT signaling pathway, Toll-like receptor signaling pathway, TNF signaling pathway. After hierarchical clustering, we identified 25 genes involved in the antiviral signaling pathway. These data suggested that *kmo* knockout might reduce the antiviral function by regulating IFN-I production and related signaling pathways (S9 Fig, Line 273-282).**

*2. Does* *QUIN have rescue effects on KMO-/- cell lines or KMO-/- mice infected with the virus?*

**Answer: Thank you for your questions.** **We conducted additional experiments to address this issue. In brief, QUIN had rescue effects on both *kmo*^-/-^ cell lines in vitro (S1 Fig, Line 136-137) and *kmo*^-/-^ mice in vivo (S10 Fig, Line 283-291).**

*3. Fig S5 provided some vital information regarding the immune dysfunction status in kmo-/- mice compared to wild mice, and thus this figure should be moved to the Text.*

**Answer: Thank you for your suggestion, and we agree with you. We now have moved this figure to the Text.**

*4. Line 269-271 and Line 285-288: repeated descriptions*

**Answer: Thank you for your comments, we have revised these descriptions, and a native English speaker has carefully edited the revised manuscript.**

*5. Line 288-290: The cited literature seems not related to the topic what authors meant*

**Answer: Thank you for your comments. We have adjusted these descriptions and cited the related literature (line 324-329).**

*6. phrases such as in vitro, in vivo, and KMO (or kmo) is sometimes in italics and sometimes not in italics*

**Answer: Thank you for your comments, we have revised it accordingly, and a native English speaker has carefully edited the revised manuscript. Thank you.**

*Reviewer #2: Demonstrate that all antiviral activities is independent of IDO-1 expression. If IDO-1 expression confound with the finding, does pharmacological inhibition of* *IDO-1 (e.g. 1-MT) still confer antiviral activity by KMO?*

**Answer: Thank you for your suggestions. As mentioned above, we have conducted additional experiments to clarify that KMO and QUIN have antiviral activities independent of IDO1 (S7 Fig, Line 211-217). In brief, we treated cells with 1-Methyl-D-tryptophan(1-MT), an inhibitor of IDO1. We found that 1-MT treatment weakened the antiviral effect by inhibiting IDO1. However, KMO and QUIN treatment could rescue the antiviral effect even after the IDO1 inhibition by 1-MT, indicating that the antiviral effects of KMO and QUIN were independent on IDO1. Thank you for your nice suggestions.**

*Reviewer #3: 1. The antiviral effect shown in Fig. 4 is very moderate, and in most case less than 1 log viral reduction was observed. It is also not shown whether the antiviral effect is cell type dependent. For example, does QUIN inhibit influenza virus replication in epithelial cells?*

**Answer: Thank you for your comments. Our data showed that KMO/QUIN exerted an unreported function to broadly inhibit DNA viruses and RNA viruses, including HSV-1, adenovirus, VSV, and influenza virus (PR8), ZIKV DENV, and SARS-CoV-2, etc. We agree with you that the antiviral effects on different viruses were different, but there was a strong inhibitory effect on some viruses, such as influenza PR8 ZIKA. Following your suggestion, we conducted additional experiments to show that QUIN has broad antiviral activity in various cells, including human lung alveolar basal epithelial cell line (A549), Bone marrow-derived macrophages (BMM) cell line, and human embryonic kidney cell line (293T) (Fig 4, and S6 Fig). Thank you.**

*2. NMDA receptor is found in neurons. However, not all the viruses studied in this ms including HSV-1, VSV, PR8, ZIKA, Dengue, and SARS-CoV-2 are neurotropic viruses. Therefore there is a lack of physiological relevance for the use of NMDA activator as antivirals. Are the cell lines used for the antiviral assay all express NMDAR? If yes, what are the evidences?*

**Answer: Thank you for your mention. Our data indicated that KMO/QUIN promoted IFN-β production by enhancing IRF3 phosphorylation, and then we tried to explore further how KMO/QUIN modulated the IRF3 phosphorylation.** **Since QUIN was previously reported as an agonist of NMDAR to induce the Ca^2+^ influx, we explore this pathway. Following your suggestions, we have validated the NMDAR expression in various cell lines in our study, using the mouse brain protein lysate as a positive control (S4 Fig, Line175-176). Thank you.**

*3. The claim of repurposing quinolinic acid as a broad-spectrum antiviral is misleading. Quinolinic acid (QUIN) is a neuroactive metabolite, and can induce oxidative neurotoxicity. High quinolinic acid levels have been associated with Alzheimer's disease, anxiety, depression, epilepsy, and Huntington's disease. Therefore, a section should be added to state the well-known side effects of quinolinic acid so the readers are not biased.
Ref:Davis I & Liu A (2015) What is the tryptophan kynurenine pathway and why is it important to neurotherapeutics? Expert Rev Neurother 15, 719–721.*

**Answer: Thank you for your kind mention. As for the potential safety of KMO and QUIN, we agree with you that this is an issue. Accordingly, we have discussed this concern and cited the related literature in our revised manuscript (line 363-370). Thank you.**

*4. In the in vivo study, QUIN is dosed by IP injection, and it has been shown that QUIN could not pass the BBB, therefore, how could it elicit the antiviral effect? A relatively low dose of* *2.5mg/kg of QUIN was used, is this sufficient to achieve the in vivo drug concentration that is needed to suppress viral infection?*

**Answer: Thank you for your questions. In our study, we administrated the QUIN in the mice experiment by intraperitoneal injection. We found that QUIN administration effectively protected mice and significantly improved survival when challenged with the highly pathogenic HSV-1 McKrae strain. In contrast, all of the mice in the vehicle group succumbed to this lethal challenge. As mentioned above, our data indicated that KMO/QUIN exerted a broadly antiviral effect by promoting the IFN-β production both *in vitro* and *in vivo*. Nevertheless, the underlying mechanisms for this novel antiviral function should be further studied.**

**We used the QUIN doses of 0.5 and 2.5mg/kg to treat HSV-infected *kmo*^+/+^ and *kmo*^-/-^ mice for the second question. Our study demonstrated that QUIN administration effectively protected the mice and reduced disease scores in a dose-dependent manner (Fig 5, and S10 Fig). The viral copies in the eye washing fluid from QUIN-treated (2.5 mg/kg) *kmo*^+/+^ and *kmo*^-/-^ mice were significantly reduced than that of mock-treated mice. Of note, in the *kmo*^+/+^ group, the viral copies were significantly reduced in the QUIN-treated group at a lower dose of 1 mg/kg (S10d Fig). In addition, IFN-βsecretion was significantly increased in the serum of QUIN-treated mice. We have mentioned these results in the revised manuscript (line 283-291).**

*5.* *NMDAR antagonists such as MK-801 have been shown to inhibit Japanese encephalitis virus (JEV)-mediated neuropathogenesis (ref below). In addition, NMDAR antagonists were also reported as VSV and influenza antivirals. Therefore, modulation of NMDAR might be a double-edge sword.
Ref: Journal of Neuroinflammation volume 15, Article number: 238 (2018)
Bioorg Med Chem. 2012 Jan 15;20(2):942-8.*

**Answer: Thank you for your kind mention. We have carefully read these articles and discussed that the modulation of NMDAR in different cells or tissues might be a double-edged sword (line 338-346). Thank you.**

**Part III – Minor Issues: Editorial and Data Presentation Modifications**
*Reviewer #1: The language needs professional editing, here are a few examples.
Line 21: replace "through kynurenine pathway" with "**through the kynurenine pathway"
Line 43: replace "mechanism" with "mechanisms"
Line 44: replace "in vivo" with italics "in vivo"
Line 83:replace "infection" with "infections"
Line 118: replace "overexpression" with "the overexpression"
Line 271: remove the space after "kmo+/+ mice"
Line 202, 204, 225, 319: replace "viral infection" with "viral infections"*

**Answer: Thank you for your** **corrections. A native English speaker has carefully edited the revised manuscript.**

*Reviewer #2: The overall narrative of the introduction and discussion that links KMO to cancer and neurodegeneration seems slightly out of context with what the study is trying to demonstrate. Hence, the direct focus of the role of KP in immunology and virology should be discussed more thoroughly. Some earlier work on KP (especially IDO-1) and antiviral is missing such as O Adams et al (as mentioned above), Gaeling L et al (2016) - https://doi.org/10.1111/febs.13966, Fernandez-Pol (2001) - https://pubmed.ncbi.nlm.nih.gov/11911246/. The authors may also want to consider linking some of the KP discoveries performed in COVID-19. See this paper: https://doi.org/10.1016/j.cels.2020.10.003.

Another question that is worth discussing in the paper is that if the host uses KMO-mediated QA to suppress viral activity, is there a long-term consequence where increased QA production chronically can promote neurodegeneration?*

**Answer: Thank you for your meaningful reminders. We agree with you and accordingly added this literature about the role of KP in immunology and virology (line 63-73, line 308-314, and line 371-376).**

**As for the potential safety of KMO and QUIN, we agree with you that this is an issue, and we have discussed this concern accordingly in our revised manuscript (line 363-370). Since QUIN cannot penetrate the blood-brain barrier, and thus QUIN was injected intraperitoneally to exert antiviral effects in our in vivo experiment to avoid the potential side effect. Nevertheless, the safety of the long-term administration of QUIN should be further monitored in the future. Thank you.**

**Altogether, all of the above comments and suggestions are very helpful in improving our manuscript. We have taken these comments into account seriously and try our best to address these questions point-by-point. After making extensive revisions, we now submit this revised manuscript, and we believe this version has been improved substantially. Thank you again for all of your comments and suggestions.**
